# Leptin Receptor Metabolism Disorder in Primary Chondrocytes from Adolescent Idiopathic Scoliosis Girls

**DOI:** 10.3390/ijms17071160

**Published:** 2016-07-20

**Authors:** Yun-Jia Wang, Hong-Gui Yu, Zhen-Hai Zhou, Qiang Guo, Long-Jie Wang, Hong-Qi Zhang

**Affiliations:** Department of Spine Surgery, Xiangya Hospital, Central South University, No. 87, Xiangya Road, Changsha 410008, China; philar1213@csu.edu.cn (Y.-J.W.); hixiatiantian@126.com (H.-G.Y.); Dreamcastlove@126.com (Z.-H.Z.); guoyisen1207@126.com (Q.G.); wlj2096@163.com (L.-J.W.)

**Keywords:** leptin, Ob-R, adolescent idiopathic scoliosis, pathogenesis

## Abstract

To investigate the underlying mechanisms of low metabolic activity of primary chondrocytes obtained from girls with adolescent idiopathic scoliosis (AIS); AIS is a spine-deforming disease that often occurs in girls. AIS is associated with a lower bone mass than that of healthy individuals and osteopenia. Leptin was shown to play an important role in bone growth. It can also regulate the function of chondrocytes. Changes in leptin and Ob-R levels in AIS patients have been reported in several studies. The underlying mechanisms between the dysfunction of peripheral leptin signaling and abnormal chondrocytes remain unclear; The following parameters were evaluated in AIS patients and the control groups: total serum leptin levels; Ob-R expression in the plasma membrane of primary chondrocytes; JAK2 and STAT3 phosphorylation status. Then, we inhibited the lysosome and proteasome and knocked down clathrin heavy chain (CHC) expression in primary chondrocytes isolated from girls with AIS and evaluated Ob-R expression. We investigated the effects of leptin combined with a lysosome inhibitor or CHC knockdown in primary chondrocytes obtained from AIS patients; Compared with the controls, AIS patients showed similar total serum leptin levels, reduced JAK2 and STAT3 phosphorylation, and decreased cartilage matrix synthesis in the facet joint. Lower metabolic activity and lower membrane expression of Ob-R were observed in primary chondrocytes from the AIS group than in the controls. Lysosome inhibition increased the total Ob-R content but had no effect on the membrane expression of Ob-R or leptin’s effects on AIS primary chondrocytes. CHC knockdown upregulated the membrane Ob-R levels and enhanced leptin’s effects on AIS primary chondrocytes; The underlying mechanism of chondrocytes that are hyposensitive to leptin in some girls with AIS is low plasma membrane Ob-R expression that results from an imbalance between the rate of receptor endocytosis and the insertion of newly synthesized receptors into the membrane.

## 1. Introduction

Adolescent idiopathic scoliosis (AIS) is a spine-deforming disease with an approximately 5% incidence rate [1]. There is no widely agreed cause of AIS. Several theories on the etiology of AIS strongly suggest that the occurrence of AIS might involve hormonal, metabolic, neuromuscular, growth-related, and genetic abnormalities [2]. One theory is that scoliosis mainly results from relative anterior spinal over-growth (RASO) with uncoupled neuro-osseous growth [3]. Previous studies have shown overgrowth of the spinal anterior vertebral column with a relatively short spinal cord [4]. Moreover, low body weights, low body mass indexes (BMI), low bone masses, and osteopenia have been documented in AIS patients [5,6], which may correlate with growth factor disturbances. Overall, AIS might be a systemic disease, and scoliosis is one of the abnormal growth patterns.

Leptin is an essential peptide hormone controlling energy homeostasis [7], particularly in regulating food intake and energy expenditure [8]. Recently, leptin was found to play a major role in bone metabolism and mediates bone growth in central and peripheral pathways [9,10]. Leptin is primarily secreted by adipocytes, and leptin receptor (Ob-R) can be detected in chondrocytes and osteoblasts [11]. Leptin regulates the osteogenic differentiation of bone marrow stem cells and the function of chondrocytes by directly binding to Ob-R [12]. Previous study showed that leptin has the following functions: promotes chondrocyte proliferation and differentiation [13]; regulates chondrocyte function by enhancing the production of collagen [14], matrix metalloproteinase (MMP), and bone morphogenetic protein (BMP) [15]; remodels the cytoskeleton [16]. Several studies have documented disorders in leptin levels in AIS patients [17,18]. Low Ob-R levels in induced AIS osteoblasts, which may lead to leptin hyposensitivity, was reported in a study performed by Liang et al. [19]. However, the relationship between dysfunctional peripheral leptin signaling and abnormal spine growth remains unclear. Hence, more investigations on the underlying mechanisms are needed.

In the present study, we hypothesized that the low membrane expression of Ob-R may be associated with the pathogenesis of AIS. The role of Ob-R level in determining chondrocyte function was examined, and the underlying mechanism of abnormal Ob-R metabolism was also studied.

## 2. Results

### 2.1. Chondrocytes Isolated from the Facet Joint of AIS Patients Showed Low Metabolic Activity

**Figure 1:**
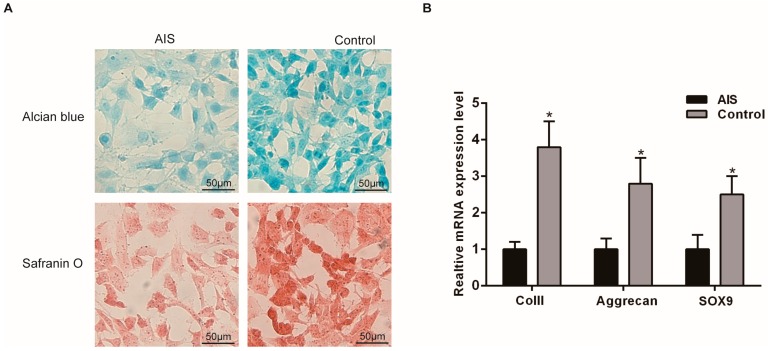
Differences in histochemical staining and the expression of chondrogenic marker genes in primary chondrocytes isolated from the AIS and control groups. (**A**) Glycosaminoglycans (GAGs) and proteoglycan content were visualized by Alcian blue and Safranin O staining, respectively. The control group showed stronger staining than the AIS group. Scale bars = 50 μm; (**B**) Total RNAs were extracted from the two groups. The relative mRNA expression levels of chondrogenic marker genes, including ColII, Aggrecan and Sox9, were detected by quantitative real-time PCR. The *Y* axis represents the fold change in transcript levels in the control and the AIS groups. The AIS group was set to 1.0. The data are displayed as the means ± SDs from 3 experiments. * *p* < 0.05 vs. the AIS group.

Human facet joints were obtained from the AIS and the control group during surgery, and primary chondrocytes were isolated from the cartilage tissue. The chondrocytes were identified at passage 3. All cultures were shown chondrocytes proved by positive toluidine blue staining and ColII immunofluorescence (Figure S1). Upon examination by inverted phase contrast microscopy, the primary cells from the AIS group showed no obvious morphological differences from the control group. Alcian blue and Safranin O staining showed less accumulation of glycosaminoglycans (GAGs) and proteoglycans in the AIS group (Figure 1A). To examine the expression of chondrogenic marker genes in the chondrocytes from the two groups, we performed RT-PCR for ColII, Aggrecan, and Sox9. The results showed that ColII, Aggrecan, and Sox9 expression was significantly reduced in the AIS group (Figure 1B).

### 2.2. AIS Patients Showed Similar Total Serum Leptin Levels to those of the Controls but Lower Membrane Ob-R Expression in Primary Chondrocytes

**Figure 2:**
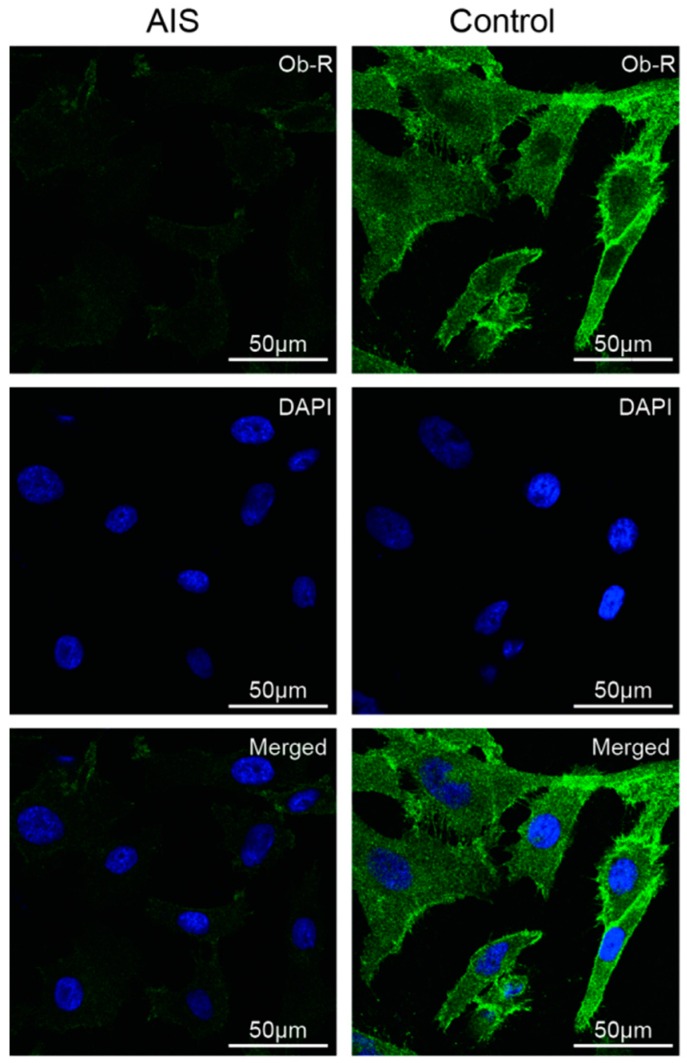
Immunofluorescence detection of Ob-R in primary chondrocytes isolated from the AIS and the control groups. Ob-R was labeled with green fluorescence, and the nuclei were stained with DAPI and shown as blue fluorescence. The AIS group is presented in the left column, and the control group is presented in the right column. Strong signals of Ob-R are present in the control group. Scale bars = 50 μm.

To assess the total serum leptin levels, 31 girls with AIS aged between 10 and 16 years and 15 non-AIS age-matched girls were recruited, and their total serum leptin levels were determined using an ELISA kit. The results and other anthropometric data of the AIS and healthy patients are listed in Table 1. The girls with AIS had significantly reduced body weights (40.90 ± 4.56 vs. 44.87 ± 4.47 kg, *p* = 0.008) and BMIs (17.66 ± 1.15 vs. 19.66 ± 0.91 kg/m^2^, *p* < 0.001). The ages, body heights, and total serum leptin levels (7.62 ± 2.80 vs. 8.89 ± 4.15 ng/mL, *p* = 0.294) were similar between the two groups. The Ob-R levels in the primary chondrocytes were determined by immunofluorescence. The AIS group showed a lower Ob-R signal than the controls (Figure 2).

### 2.3. Chondrocytes of AIS Girls Showed Reduced JAK2 and STAT3 Phosphorylation

**Figure 3:**
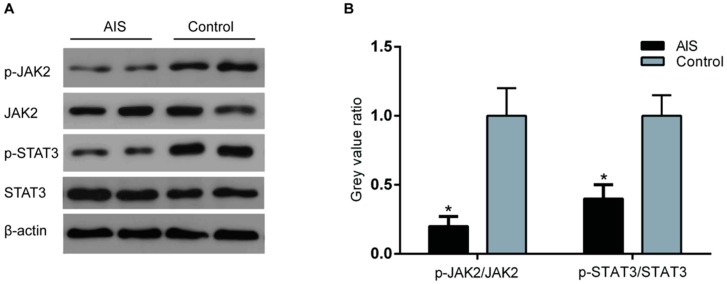
JAK2 and STAT3 phosphorylation status in the AIS and the control groups. (**A**) Protein samples were acquired from the cartilage tissue of the participants in both the AIS and the control groups. The expression of p-JAK2, total JAK2, p-STAT3, and total STAT3 was analyzed by Western blotting. β-actin was used as an internal reference. Representative Western blots from three independent experiments are shown; (**B**) Relative quantitation of the p-JAK2/JAK2 and p-STAT3/STAT3 levels is shown in the graphs, and the mean value for the control groups is 1.0. The *Y* axis represents the fold change between the AIS and control groups. * *p* < 0.05 vs. the controls.

To determine the activation level of the major leptin signaling pathway—the JAK2/STAT3 signaling pathway—in AIS patients, we obtained the facet joints during surgery, and extracted the protein samples from the cartilage tissue of the facet joint. JAK2 and STAT3 expression and phosphorylation (two main participants in the JAK2/STAT3 signaling pathway) were detected by Western blotting. The results showed a reduction in the p-JAK2 and p-STAT3 levels in the AIS patients, but JAK2 and STAT3 expression was not significantly different between the two groups (Figure 3).

### 2.4. Lysosome Inhibition Increases the Ob-R Content but Has No Effect on Ob-R Membrane Expression or Leptin’s Effects on AIS Primary Chondrocytes

**Figure 4:**
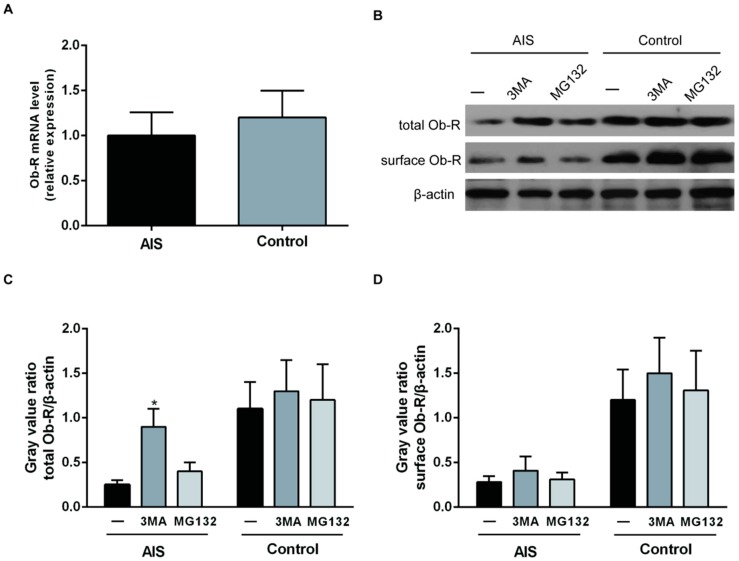
Effect of the lysosome inhibitor 3MA on Ob-R expression in chondrocytes from the AIS and control groups. RNA and protein samples were extracted from primary chondrocytes from the participants in both the AIS and the control groups. (**A**) RT-PCR was used to detect the Ob-R mRNA expression levels. The *Y* axis represents the fold change in transcript levels in the AIS and control groups. The AIS group was set to 1.0. The data are displayed as the means ± SDs from 3 experiments; (**B**) Chondrocytes from the AIS and the control groups were incubated with the lysosome inhibitor 3MA (5 mM) or proteasome inhibitor MG132 (2 μM) for 3 h. Total and membrane proteins were extracted. Ob-R expression was analyzed by Western blotting. β-actin was used as an internal reference. Representative Western blots from three independent experiments are shown; (**C**) The quantitation of the total Ob-R/β-actin levels is shown in the graphs, and the mean value for the untreated control groups was set to 1.0. The *Y* axis represents the fold change between the AIS and control groups. * *p* < 0.05 vs. the untreated AIS; and (**D**) The quantitation of the surface Ob-R/β-actin levels is shown in the graphs, and the mean value of the untreated control groups was set to 1.0. The *Y* axis represents the fold change between the AIS and control groups.

**Figure 5:**
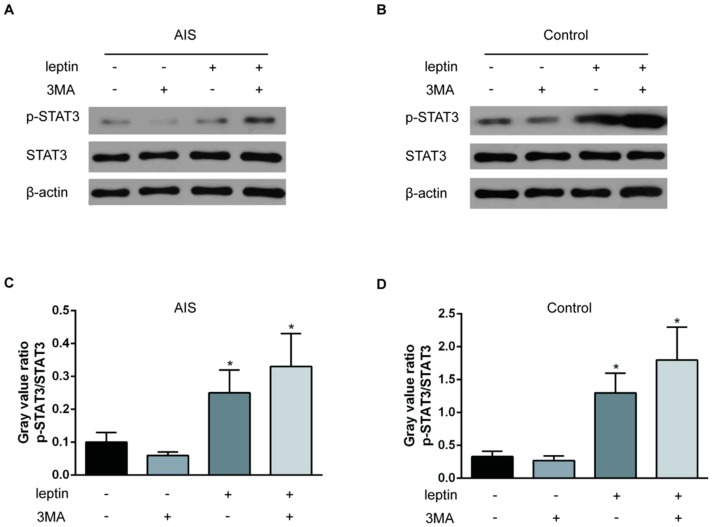
Effect of leptin and 3MA on the STAT3 phosphorylation status in chondrocytes from the AIS and control groups. (**A**,**B**) Chondrocytes from the AIS and control groups were incubated with leptin (10 ng/mL) for 5 h or the lysosome inhibitor 3MA (5 mM) for 3 h. Total proteins was extracted. The expression of p-STAT3 and STAT3 was analyzed by Western blotting. β-actin was used as an internal reference. Representative Western blots from three independent experiments are shown; (**C**,**D**) The quantitation of the p-STAT3/STAT3 levels is shown in the graphs. The *Y* axis represents the fold change between different treatments. * *p* < 0.05 vs. the group untreated with leptin or 3MA.

To investigate the cause of the reduced Ob-R expression in the AIS primary chondrocytes, we examined Ob-R mRNA expression in the two groups. The results showed no significant differences in Ob-R mRNA expression in the two groups (Figure 4A). Then, we treated the chondrocytes with the lysosome inhibitor 3MA (5 mM) or the proteasome inhibitor MG132 (2 μM) for 3 h, and Ob-R expression was analyzed by Western blotting. 3MA only increased the total Ob-R content in the AIS group, without influencing the membrane Ob-R content. MG132 treatment had no effect on Ob-R expression in the two groups (Figure 4B–D). To verify the effect of 3MA on the Ob-R content in the membrane, the cells were treated with either leptin (10 ng/mL) for 5 h or 3MA (5 mM) for 3 h or with both compounds. Leptin induced STAT3 phosphorylation in the two groups, whereas the 3MA treatment did not enhance leptin’s effect on STAT3 phosphorylation in the two groups (Figure 5).

### 2.5. CHC Knockdown Upregulates the Membrane Ob-R Levels

**Figure 6:**
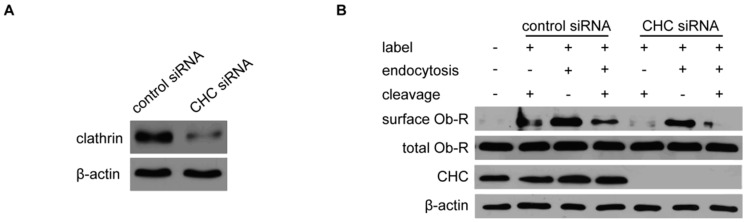

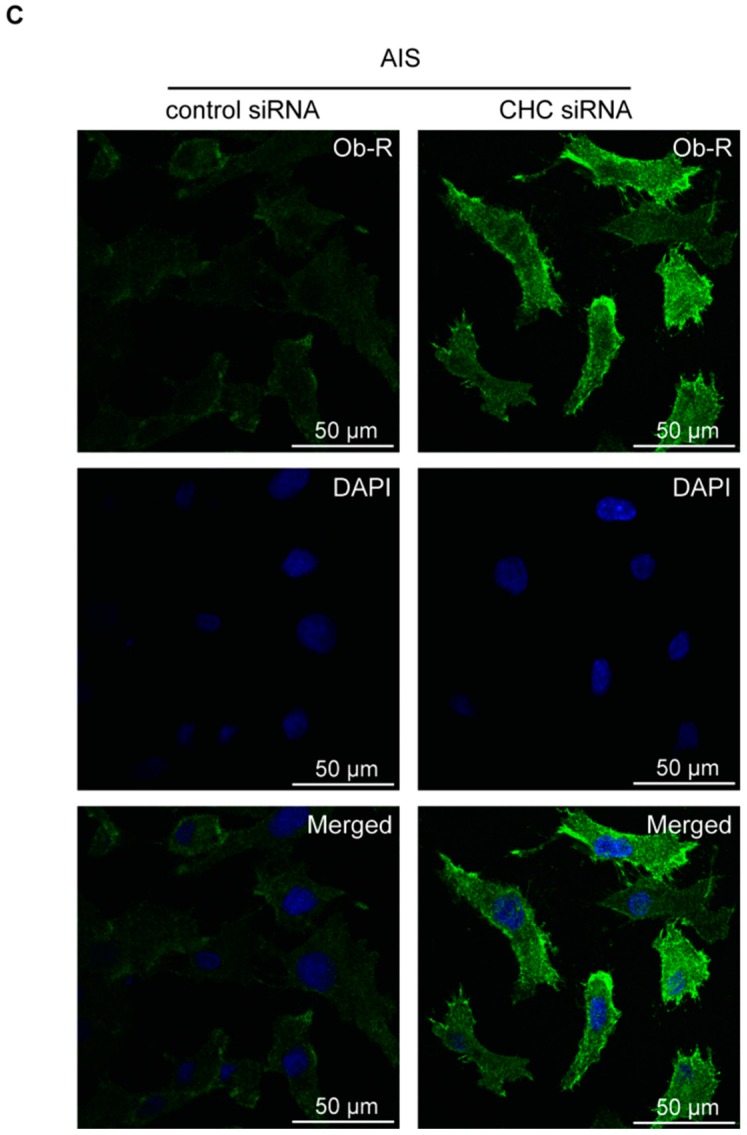
CHC knockdown upregulates Ob-R expression in the membrane of chondrocytes from AIS patients. (**A**) The efficiency of CHC knockdown was evaluated by Western blotting, and β-actin was used as an internal reference; (**B**) Chondrocytes from AIS patients were transfected with the control siRNA or CHC siRNA, followed by biotinylation of membrane protein. Then, the endocytosis of the biotinylated plasma membrane protein was induced with/without l-glutathione (GSH) buffer treatment. Alternatively, the cells were treated with GSH buffer but did not undergo the endocytosis procedure. The total and biotinylated plasma membrane proteins were extracted. Ob-R expression was analyzed by Western blotting. β-actin was used as an internal reference. Representative Western blots from three independent experiments are shown; (**C**) Chondrocytes from AIS patients were transfected with the control siRNA or CHC siRNA. Ob-R was labeled with green fluorescence, and the nuclei were stained with DAPI and shown as blue fluorescence. Strong signals of Ob-R were observed in the cells transfected with the CHC siRNA. Scale bars = 50 μm.

Because the inhibition of Ob-R lysosomal degradation had no effect on Ob-R cell surface expression, we hypothesized that inadequate endocytosis of Ob-R may accelerate the rate of Ob-R internalization in AIS primary chondrocytes. To investigate whether endocytosis affects the membrane expression of Ob-R, the expression of CHC was inhibited by siRNA transfection in primary chondrocytes from the AIS group (Figure 6A). The cell surface protein were biotinylated to elucidate the contribution of CHC to Ob-R expression. CHC knockdown led to a significant increase in the membrane expression of Ob-R, but it did not increase total Ob-R expression (Figure 6B). The effects of CHC knockdown were confirmed by immunofluorescence staining of AIS primary chondrocytes after CHC siRNA transfection; strong signals of Ob-R were observed in the membrane (Figure 6C).

### 2.6. CHC Knockdown Enhances Leptin’s Effects on AIS Primary Chondrocytes

**Figure 7:**
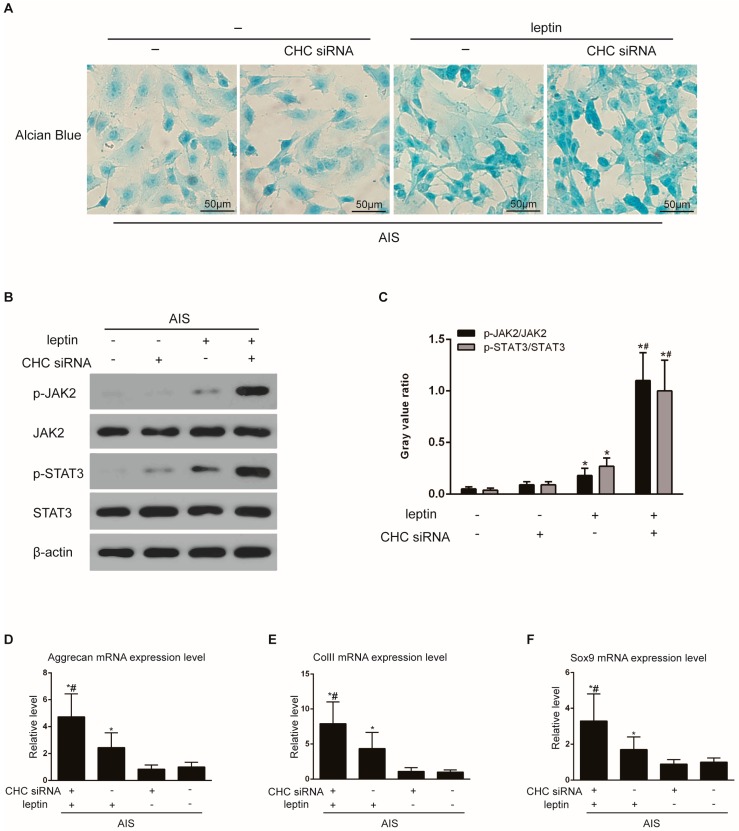
CHC knockdown enhances leptin’s effect on the chondrocytes from AIS patients. Chondrocytes from the AIS group were transfected with the control siRNA or CHC siRNA, followed by treatment with or without leptin (10 ng/mL) for 5 h. (**A**) The GAG content was visualized by Alcian blue staining. Stronger Alcian blue staining was observed in the leptin+CHC siRNA group than in the other group. Scale bars = 50 μm; (**B**) Total proteins were extracted. The expression of p-JAK2, total JAK2, p-STAT3, and total STAT3 was analyzed by Western blotting. β-actin was used as an internal reference. Representative Western blots from three independent experiments are shown; (**C**) The relative quantitation of the p-JAK2/JAK2 and p-STAT3/STAT3 levels is shown in the graphs. * *p* < 0.05 vs. the group untreated with leptin or CHC siRNA; ^#^ *p* < 0.05 vs. the leptin-treated group; (**D**–**F**) Total RNAs were extracted from the chondrocytes. The relative mRNA expression of chondrogenic marker genes, including Aggrecan, ColII, and Sox9, was detected by quantitative real-time PCR. The *Y* axis represents the relative fold change in the transcript levels in the four groups. The untreated control group was set to 1.0. The data are displayed as the means ± SDs from 3 experiments. * *p* < 0.05 vs. the group untreated with leptin or CHC siRNA; ^#^ *p* < 0.05 vs. the leptin-treated group.

Finally, we examined whether CHC knockdown affects the AIS primary chondrocytes’ reactivity to leptin. After combining CHC siRNA transfection with leptin treatment, the expression of GAGs, p-JAK2 and p-STAT3 proteins, and Aggrecan, ColII, and Sox9 mRNAs were examined. As expected, chondrocytes that were transfected with the CHC siRNA and then incubated with 10 ng/mL leptin for 5 h exhibited increased GAG accumulation, JAK2 and STAT3 phosphorylation, and Aggrecan (4.73-fold), ColII (7.92-fold), and Sox9 (3.30-fold) mRNA expression levels compared with the control cells untreated with CHC siRNA or leptin. (Figure 7).

## 3. Discussion

Adolescent idiopathic scoliosis is a spine-deforming disease characterized by three-dimensional deformation of the spine and an approximately 5% incidence rate. The pathogenesis of AIS remains poorly understood. It is suggested that both genetic and biological abnormalities could have impacts on spine growth. The hypothesis of an imbalance in the growth of the anterior and posterior column of the spine during adolescence has been documented in a previous study [20]. Increased chondrocyte activity in the anterior column was observed in AIS patients. In the present study, human facet joints were obtained from the AIS and the control group during surgery, and primary chondrocytes were isolated from the cartilage tissues. We observed decreased cartilage matrix synthesis in the AIS group, which may be one reason for the asymmetric spine growth.

Girls with AIS have low BMIs and deranged bone masses, suggesting that AIS is a systemic disease that may result from metabolism-endocrine disorders [21]. In previous studies, decreased leptin levels [22], defects in the leptin signaling pathway, leptin hyposensitivity, and high leptin bioavailability were reported in girls with AIS [19]. Leptin modulates pubertal growth, bone formation, and energy metabolism [23]. In Burwell’s hypothesis [21], altered leptin sensitivity resulting in increased sympathetic nervous system (SNS) activity may contribute to osteopenia, skeletal overgrowth, and spinal skeletal length asymmetries. In our study, the total serum leptin levels were similar between the AIS and control group, which is in accordance with an investigation performed by Liang et al. [19]. However, lighter body weights of the AIS group were documented in the present study, which may have an impact on the results. It is well-known that serum leptin binding to Ob-R activates downstream pathways [24]. In primary human chondrocytes, leptin enhances chondrocyte proliferation and the subsequent cell differentiation and increases collagen II expression by leptin-enhanced BMP-2 synthesis [15]. In our study, we found that the primary chondrocytes from the AIS group showed lower membrane Ob-R expression than the controls, which may lead to leptin hyposensitivity in chondrocytes. Decreased activation of the JAK2/STAT3 pathway—the major pathway activated by leptin [25]—was observed in the facet joints from the AIS patients. The results support the hypothesis that abnormal membrane Ob-R levels could led to the chondrocyte dysfunction in the posterior column of the spine in girls with AIS.

Our results showed no significant differences in Ob-R mRNA expression between the AIS and the control groups, suggesting that the low membrane expression of Ob-R may result from abnormal Ob-R metabolism at the post-translational level. Ob-R is a single membrane-spanning protein of the class I cytokine receptor family [26]. Several isoforms of Ob-R, including Ob-Ra, Ob-Rc, Ob-Rd (short isoforms), and Ob-Rb (long isoform), were identified as mRNA splice variants [27]. They differed in the C-terminus of their cytoplasmic tails. The long form Ob-Rb is the major receptor of leptin by activating the downstream JAK-STAT signaling pathway. Ob-R is mainly localized in the cytoplasm rather than on the cell surface [28]. After Ob-R is synthesized, approximately 50% of the receptors are retained in the Golgi complex through their transmembrane domains [29]. Surface localized Ob-R is constitutively endocytosed through the clathrin-dependent pathway and degraded in lysosomes without recycling to the membrane [30,31]. The internalization process is mediated by the cytoplasmic tail of Ob-R. Nevertheless, because membrane-localized Ob-R determines cellular leptin sensitivity [32], any factors that regulate the intracellular trafficking or internalization of Ob-R could alter leptin sensitivity. We found that lysosome inhibition could increase total Ob-R expression but had no effect on the membrane Ob-R level in primary chondrocytes from AIS patients. CHC knockdown increased Ob-R cell surface expression and enhanced leptin’s effects on AIS primary chondrocytes. The results suggest that an imbalance between the rate of endocytosis and the insertion of newly synthesized receptors into the membrane leads to low plasma membrane Ob-R expression in primary chondrocytes from the facet joints from AIS patients. However, it is not clear whether endocytosis or receptor transport to the cell surface is critical for this disorder, and the underlying mechanisms require further investigation.

Our study has some limitations. One of them is the limited sample size of the recruited patients and the relatively narrow spectrum of inclusion criteria. Thus, we could not investigate the leptin levels and Ob-R expression in primary chondrocytes from different genders and body types, and patients with lower Cobb angles were excluded because they do not require surgical therapy. Another limitation is that the primary chondrocytes were extracted from only the facet joint, and the results of our study may not represent general trends. Another limitation is that the serum level of melatonin, estrogen and ghrelin were not evaluated, which may affect leptin level. Thus, the differences must be studied further in other chondrocytes.

In conclusion, our findings provide new insights into the pathogenesis of AIS. We found that the chondrocytes from some AIS patients were hyposensitive to leptin, and the underlying mechanism is low plasma membrane Ob-R expression resulting from an imbalance between the rate of endocytosis and the insertion of newly synthesized receptors into the membrane. The low membrane Ob-R level may be a biomarker of progressive scoliosis which need surgical intervention. Moreover, the present study provides new therapeutic insights into this complex disorder.

## 4. Materials and Methods

### 4.1. Ethics Statement

This study was approved by the Ethics Committee of Xiangya Hospital of Central South University (Changsha, China). Procedures involving human subjects were performed in accordance with The Code of Ethics of the World Medical Association (Declaration of Helsinki). Informed consent was obtained from all patients and/or their parents prior to inclusion in the study.

### 4.2. Reagents

Recombinant human leptin was purchased from PeproTech (Rocky Hill, NJ, USA). The rabbit polyclonal antibody against collagen II (ab34712), mouse monoclonal antibody against the leptin receptor (ab2143), and rabbit polyclonal antibody against beta-actin (ab8227) were obtained from Abcam (Cambridge, MA, USA). The chicken anti-rabbit IgG (H+L) secondary antibody (A-21442), chicken anti-mouse IgG (H+L) secondary antibody (A-21200), Sulfo-NHS-SS-Biotin (21331), and avidin agarose (20219) were purchased from Thermo Fisher Scientific (Waltham, MA, USA). The rabbit monoclonal antibody against JAK2, rabbit monoclonal antibody against p-JAK2 (Tyr1008), mouse monoclonal antibody against STAT3, rabbit monoclonal antibody against p-STAT3 (Ser727) and rabbit monoclonal antibody against CHC were purchased from Cell Signaling Technology (Beverly, MA, USA). Toluidine blue (6586-04-5), Safranin O (477-73-6), Alcian blue (33864-99-2), 3-methyladenine (5142-23-4), and MG-132(R) (1211877-36-9) were purchased from Sigma-Aldrich (St. Louis, MO, USA). The Human Leptin ELISA kit was obtained from R&D Systems (Minneapolis, MN, USA).

### 4.3. Patients and Samples

Thirty-one girls with AIS aged between 10 and 16 years were recruited from our hospital. The diagnosis of AIS was confirmed by clinical and radiological findings using the standard standing antero-posterior radiography method. The inclusion criterion was a maximum Cobb angle of more than 10°. Patients with congenital vertebral malformations, neuromuscular diseases, metabolic diseases, skeletal dysplasia, connective tissue abnormalities, mental retardation, a history of recent steroid intake, or other diseases affecting bone metabolism were excluded from the study. The control group, which included fifteen non-AIS age-matched girls, was examined to eliminate any spinal deformity. To avoid bias, subjects with BMIs >23.0 kg/m^2^ (according to overweight criteria classified by World Health Organization for the Asian populations) were excluded because obese individuals are known to have abnormal leptin levels. Peripheral venous blood samples were collected in the morning (between 7:00 and 9:00 a.m.) from all subjects in the AIS and control group after an overnight fast, and the sera were separated and stored at −80 °C until analyses.

### 4.4. Isolation and Culture of Human Primary Chondrocytes

A total of 15 facet joints were harvested from the apical areas of fifteen AIS patients undergoing posterior spinal fusion; the control group included 8 facet joints harvested from eight age-matched girls with thoracolumbar spinal injuries or lumbar disc herniation or spinal tuberculosis undergoing spinal surgical treatment. Briefly, the cartilage from the facet joint tissue was peeled and cut into pieces with a sterile scalpel, followed by 0.25% collagenase type II (Sigma-Aldrich, St. Louis, MO, USA) treatment overnight at 37 °C. The supernatant was removed after centrifugation, and the pellet was resuspended in 0.05% trypsin (Gibco, Carlsbad, CA, USA) for 3 h. After passing through a cell strainer, the cells were collected, plated at a density of 2 × 10^4^ cells/cm^2^ in DMEM/Nutrient Mixture Ham’s F-12 (DMEM/F12 1:1) medium (HyClone, Logan, UT, USA), supplemented with 10% fetal bovine serum (Gibco, Carlsbad, CA, USA), and cultured at 37 °C and 5% CO_2_ for two passages before use.

### 4.5. Cell Staining

Chondrocytes were plated in 24-well plates with coverslips (Lab-Tek, Nalge Nunc International, Roskilde, Denmark) and allowed to reach 80% confluence before processing. The cells were fixed with 4% Paraformaldehyde (PFA) for 20 min and washed three times with phosphate-buffered saline (PBS). For toluidine blue staining, the cells were incubated with 0.05% toluidine blue (in 100 mM Tris buffer, pH 8) for 1 h. For Alcian blue staining, the cells were incubated with 1% Alcian blue 8GX (in 1% acetic acid) for 30 min. For Safranin O staining, the cells were incubated with 1% fast green (in 1% acetic acid) for 5 min and then stained with 0.1% Safranin O (in distilled water) for 20 min. The excess dye was washed out with sterile, distilled deionized water. All staining procedures were stopped with two washes in 70% ethanol and three washes in PBS; the slides were mounted with glycerol and sealed with nail polish.

### 4.6. Immunofluorescence and Confocal Microscopy

Chondrocytes from AIS patients and non-AIS patients were plated on slides placed in 24-well plates. After culturing for 24 h under standard conditions, the cells were washed twice with PBS, fixed with 4% PFA for 20 min at room temperature, permeabilized with 0.1% Triton X-100 for 10 min, and blocked with 5% BSA and 0.05% Tween 20 in PBS for 1 h. Then, the cells were incubated with the ColII (1:200) or Ob-R (1:200) primary antibodies for 1 h. After rinsing for 3 × 10 min, the cells were incubated with a fluorescently labeled secondary antibody (1:200) for 1 h at room temperature in the dark, and the nuclei were stained with 4, 6-diamidino-2-phenylindole (DAPI) (Sigma-Aldrich, St. Louis, MO, USA). Fluorescence localization was acquired using a laser-scanning microscope (FV10-ASW 1.7; Olympus, Tokyo, Japan).

### 4.7. RNA Extraction and Real-Time PCR

Total RNA was extracted from chondrocytes using TRIzol reagent (Invitrogen, Carlsbad, CA, USA). cDNAs were synthesized from total RNA using a TaqMan cDNA synthesis kit (Applied Biosystems, Foster, CA, USA). Real-time PCR was performed using SYBR Green Master Mix with rhodamine X (ROX) (Takara, Kusatsu, Shiga, Japan) and a 7900HT Fast Real-Time PCR system (Applied Biosystems, Foster, CA, USA). The primers used for quantitative PCR included the following: ColII, CTACGGTGTCAGGGCCAG (forward) and GCAAGATGAGGGCTTCCATA (reverse); Aggrecan, CTGAAGTTCTTGGAGGAGCG (forward) and CGCTCAGTGAGTTGTCATGG (reverse); Sox9, GTACCCGCACTTGCACAAC (forward) and TCTCGCTCTCGTTCAGAAGTC (reverse); and β-actin, ACTCTTCCAGCCTTCCTTCC (forward), and GTACTTGCGCTCAGGAGGAG (reverse). Each cycle was 95 °C for 5 s and 60 °C for 30 s (for 40 cycles).

### 4.8. siRNA Transfection

Chondrocytes that had reached 40%–60% confluence were transfected with the CHC siRNA or control siRNA in serum-free conditions. Oligofectamine (Invitrogen, Carlsbad, CA, USA) transfection reagents were used according to the manufacturer’s instructions. The siRNA sequences were: CHC siRNA, 5′-AACCUGCGGUCUGGAGUCAAC-3′, and non-targeting siRNA, 5′-UUCUCCGAACGUGUCACGUTT-3′. Briefly, Oligofectamine and the oligonucleotides were mixed with Opti-MEM. The mixture was incubated at room temperature for 20 min and then added to the cell cultures for 3 h. Then, the mixture was replaced with fresh medium containing serum. Total proteins were extracted from the cells transfected with the CHC siRNA and non-targeting siRNA. CHC expression was analyzed by Western blot analysis to evaluate the silencing effect.

### 4.9. ELISA

An enzyme-linked immunosorbent assay (ELISA) kit was used to measure the serum leptin levels according to the manufacturer’s recommended protocol. The lowest detectable leptin concentration was 0.50 ng/mL.

### 4.10. Membrane Protein Isolation and Western Blot Analysis

The cell surface protein biotinylation method was used to isolate the membrane protein. Briefly, after the cells reached 80% confluence, the medium was changed into a 250 μg/mL Sulfo-NHS-SS-Biotin solution and then the cells were incubated for 20 min at 4 °C; the reaction was then quenched by a Tris-HCl solution. If only the membrane protein were needed, the endocytosis procedure could be skipped, and the cells could be collected directly. To assess the endocytosis of biotinylated plasma membrane protein, the cells were cultured at 37 °C and 5% CO_2_ for 1 h after the quenching solution was replaced with fresh medium. Subsequently, the cells were cooled to 4 °C and incubated with l-glutathione (GSH) buffer for 15 min to cleave the disulfide bonds on the biotinylated protein. Then, the cells were collected and centrifuged, and the cell pellet was re-suspended with lysis buffer and sonicated. After the protein concentrations were measured, 500 μg of protein was transferred to 100 μL of prewashed streptavidin agarose and incubated for 60 min at room temperature in a tube rotator. The streptavidin agarose was centrifuged, washed three times, and incubated with 30 μL of 2× SDS-PAGE sample buffer with DTT for 60 min at room temperature in a tube rotator. After centrifugation, the supernatant was used for Western blotting.

Human facet joints were obtained after surgery, and the cartilage tissue was peeled from the facet joint using a scalpel and then sequentially extracted in buffer A (0.15 M NaCl and 50 mM Tris), buffer B (1 M NaCl, 10 mM EDTA, and 50 mM Tris) and buffer C (4 M GuHCl, 10 mM EDTA, and 50 mM Tris). The supernatants from the extractions were ethanol-precipitated and resuspended for future analysis. The total cellular proteins were collected from the cells with lysis buffer (50 mM Tris-HCl, pH 7.5, 150 mM NaCl, 1% Nonidet P-40, 0.5% sodium deoxycholate, and complete protease inhibitors) and then sonicated. After boiling at 100 °C for 5 min, the lysates were stored at −20 °C for future analysis. The protein levels were quantified using a BCA protein assay reagent (Thermo Fisher Scientific, Waltham, MA, USA). Twenty micrograms of total protein from each sample were loaded onto a 10% SDS gel and transferred to a PVDF (Polyvinylidene Difluoride) membrane (Bio-Rad, Hercules, CA, USA). After blocking with skim milk for 1 h at room temperature, the membranes were incubated with primary antibodies overnight at 4 °C on a rocker. Antibodies against the following proteins were used: p-JAK2, JAK2, p-STAT3, STAT3, Ob-R, CHC, and β-actin. After incubation with secondary antibodies in blocking buffer at room temperature for 1 h, the blots were visualized using a Chemiluminescent Protein Detection Module (Millipore, Temecula, CA, USA).

### 4.11. Statistical Analysis

Each experiment described above was repeated at least two times. The results were presented as the means ± SDs. The serum leptin levels in the AIS patients and normal controls were compared using an independent sample *t*-test. The differences between groups were assessed using Student’s *t*-test or ANOVA, and *p*-values <0.05 were considered significant.

## Figures and Tables

**Table 1:** Anthropometrics and total serum leptin levels in the AIS patients and controls.

	AIS (*n* = 31)		Controls (*n* = 15)		*t* Test	*p*
	Mean	SD	Mean	SD		
Age	12.81	1.82	13.67	1.67	−0.46	0.642
Weight	40.90	4.56	44.87	4.47	−2.78	0.008 *
Height (cm)	152.06	7.11	150.93	6.49	0.52	0.606
BMI (kg/m^2^)	17.66	1.15	19.66	0.91	−5.90	<0.001 *
Leptin levels (ng/mL)	7.62	2.80	8.89	4.15	−1.08	0.294

*: *p* < 0.05. (Independent-samples *t* test); BMIs were calculated by dividing the body weight by the squared arm span (m^2^); Abbreviations: AIS, adolescent idiopathic scoliosis; BMI, body mass index.

## Supplementary Materials

Supplementary materials can be found at http://www.mdpi.com/1422-0067/17/7/1160/s1.

## Abbreviations

Ob-R	leptin receptor
AIS	adolescent idiopathic scoliosis
BMI	body mass index
CHC	clathrin heavy chain
RASO	relative anterior spinal overgrowth
BMP	bone morphogenetic protein
MMP	matrix metalloproteinase
PFA	Paraformaldehyde
PBS	phosphate-buffered saline
DAPI	4, 6-diamidino-2-phenylindole
GSH	l-glutathione
GAGs	Glycosaminoglycans
SNS	sympathetic nervous system
PVDF	Polyvinylidene Difluoride
