# Supplementary material for: Leptin Receptor Metabolism Disorder in Primary Chondrocytes from Adolescent Idiopathic Scoliosis Girls
Source: Int J Mol Sci. 2016 Jul 20;17(7):1160. doi: 10.3390/ijms17071160 (PMC4964532; doi:10.3390/ijms17071160)
Supplement: Supplementary file 1 [file ijms-17-01160-s001.pdf]

Supplementary Materials: Leptin Receptor Metabolism Disorder in Primary Chondrocytes from Adolescent Idiopathic Scoliosis Girls

Yun-Jia Wang, Hong-Gui Yu, Zhen-Hai Zhou, Qiang Guo, Long-Jie Wang and Hong-Qi Zhang

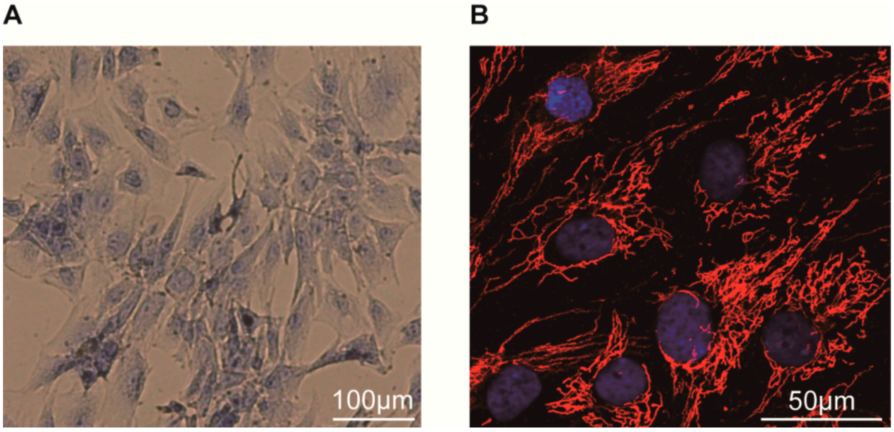

Figure S1. Identification of primary chondrocytes isolated from the human facet joint. (**A**) Cell morphology and biological characteristics were determined by toluidine blue staining. Scale bar = 100 μm; (**B**) Collagen II expression was shown by immunofluorescence staining. Scale bar = 50 μm.

Table S1. Anthropometrics and total serum leptin levels in the AIS patients.

	AIS		
	**AIS (*n* = 31)**	**Suigical (*n* = 15)**	**Non-Surgical (*n* = 16)**
	**Mean ± SD**	**Mean ± SD**	**Mean ± SD**
Age	12.81 ± 1.82	13.20 ± 1.47	12.44 ± 2.06
Weight (kg)	40.90 ± 4.56	40.93 ± 6.55	41.81 ± 3.99
Height (cm)	152.06 ± 7.11	151.07 ± 6.37	153.00 ± 7.83
BMI (kg/m^2)	17.66 ± 1.15	17.91 ± 2.46	17.84 ± 0.74
Cobb angle (°)	39.90 ± 14.95	53.47 ± 7.68	27.19 ± 5.94 *
Leptin levels (ng/mL)	7.62 ± 2.80	7.94 ± 3.87	7.69 ± 3.38

*: $p < 0.05$ vs. the suigical group (Independent-samples *t* test); BMIs were calculated by dividing the body weight by the squared arm span (m^2); Abbreviations: AIS, adolescent idiopathic scoliosis; BMI, body mass index.

Table S2. Information of the control patients (for primary chondrocytes isolated).

Patient No.	Dignosis	Age	Weight (kg)	Height (cm)	BMI (kg/m^2)	Leptin Level (ng/mL)
1	Multiple trauma	13	40	147	18.51	7.40
2	L1 fracture	14	45	150	20	10.00
3	Multiple trauma	13	38	143	18.58	6.37
4	Multiple trauma	15	47	155	19.56	8.13
5	LDH	14	44	157	17.85	8.53
6	LDH	15	52	160	20.31	9.35
7	LDH	14	49	150	21.78	6.78
8	spinal tuberculosis	13	38	148	17.35	8.13

BMIs were calculated by dividing the body weight by the squared arm span (m^2); Abbreviations: LDH, lumbar disc herniation; BMI, body mass index.
